# Microfiber evanescent-field photothermal gas detection using acoustic-induced mode-dependent frequency shift

**DOI:** 10.1515/nanoph-2023-0092

**Published:** 2023-06-23

**Authors:** Yi Zhu, Anbo Guo, Jiangtao Xu, Zhengwei Zhang, Fufei Pang, Weijian Zhang, Xianglong Zeng, Jianfeng Sun

**Affiliations:** The Key Lab of Specialty Fiber Optics and Optical Access Network, Joint International Research Laboratory of Specialty Fiber Optics and Advanced Communication, Shanghai University, Shanghai 200444, China; Shanghai Satellite Network Research Institute Co., Ltd, Shanghai, China

**Keywords:** acoustic-induced mode-dependent frequency shift, microfiber evanescent field, photothermal heterodyne gas detection, transverse modes cyclic conversion

## Abstract

In this study, we experimentally showcase the microfiber evanescent-field photothermal gas detection by exploiting all-fiber MHz-level frequency shift scheme. Based on the acousto-optic interaction effect, the low-frequency shifts of 0.9 MHz and 1.83 MHz can be obtained through the cyclic conversion between the transverse core modes LP_01_ and LP_11_ in the few-mode fiber. Our proposed all-fiber frequency shifters show flexible MHz-level up(down) frequency shifts with superior sideband rejection ratio (over 40 dB) and low insertion loss (less than 1 dB). Furthermore, an all-fiber heterodyne interferometric detection system is implemented by leveraging the above low-frequency shifters, in which around 1-μm-diameter microfiber is investigated for photothermal gas detection. A pump-probe configuration is employed to obtain the photothermal effect induced by the gas absorption of the modulated evanescent field. By demodulating the phase of the beat signal output by the interferometer, an equivalent detection limit (1*σ*) of 32 ppm and a response time of 22 s are achieved for ammonia, as well as 0.24 % instability within 48 pump cycles. Given its compact all-fiber configuration and high sensitivity with fast response, the experimental results can pave the way for widespread applications like heterodyne detection, fiber optical sensors, and interplanetary coherent communications.

## 1 Introduction

Laser absorption spectroscopic gas detection technique has been widely used in industrial manufacturing, environmental monitoring, and biomedicine [1, 2]. However, when detecting weak absorbing gases such as ammonia at the near-infrared (NIR) band, conventional approaches often necessitate the utilization of multi-pass cells [3, 4] with ultra-long optical paths to enhance the sensitivity, which is accompanied by the large-sized sensing modules, as well as the high costs of the equipment and maintenance. As a novel and flexible means, photothermal interferometry has attracted growing attention in recent years [5–7]. Absorption of the modulated pump light by gas molecules heats up the local sensing medium and then periodically changes its refractive index. This mechanism is the so-called photothermal (PT) effect which induces the phase modulation of the probe light propagating through the same medium [5, 8]. The gas information can be derived by demodulating the phase of the beat signal output by the optical interferometer. Recently, a new PT gas detection system based on the optical nano waveguide has been demonstrated [9], which dramatically reduces the response time and with a more compact structure, compared to the hollow-core fiber (HCF) approach [10]. Moreover, the heterodyne interferometry, which offers simpler configuration and high robustness, is gaining popularity in the field of PT gas detection because it eliminates the active regulating elements such as a servo-control loop [8, 11, 12].

The kernel of the conventional optical heterodyne interferometer is the frequency shifter, which is responsible for offering an optical frequency difference between the probe beam and the reference beam [13, 14]. To achieve this, an acousto-optic modulator (AOM) with bulk Bragg cell crystal is typically utilized. Although the commercially compact fiber-pigtailed AOMs are actually based on the miniatured bulk acousto-optic crystals, the ultrahigh heterodyne frequencies of tens or hundreds of MHz generated by the AOMs are still challenging to be demodulated for most commercial lock-in amplifiers (LIAs). As an alternative, the compromised solution is cascading two AOMs with slightly different frequency-shift magnitudes to obtain a low-frequency shift, which leads to high complexity with additional insertion loss [15–17]. Thus, an ultracompact all-fiber device that can provide reliable optical low-frequency shift is desirable. Additionally, incorporating this all-fiber low-frequency shift technology with microfiber evanescent-field technique is expected to further improve the integration of the PT gas detection system, facilitating its widespread application.

In the present work, we experimentally demonstrate two highly promising all-fiber acousto-optic frequency shift (AOFS) schemes, which are based on the acoustic-induced cyclic conversion between the transverse core modes propagating through the few-mode fiber (FMF) and their mode-dependent frequency-shift effect [18, 19]. When an appropriate ultrasonic vibration is exploited to form a dynamic long-period grating along the unjacketed FMF, not only the energy coupling between LP_01_ and LP_11_ core modes is enabled, but also an optical frequency shift is achieved for the converted transverse LP mode [20, 21]. By cascading the acoustic-induced fiber gratings (AIFGs) or with a long-period fiber grating (LPFG), the low-frequency shifts of 1.83 MHz and 0.9 MHz are obtained with the insertion losses of 0.83 dB and 2.05 dB, respectively. Both schemes possess frequency sideband rejection ratios exceeding 40 dB and wavelength tuning ranges wider than 200 nm. Furthermore, these two low-frequency shift schemes are applied in all-fiber PT heterodyne gas detection. Through the PT enhancement facilitated by the tapered microfiber with a diameter of 1 μm and a length of 4 mm, ammonia detection is achieved with an equivalent detection limit (1*σ*) of 32 ppm and a response time of 22 s. The experimental results exhibit the potential utility of the proposed AOFS schemes in a wide range of scenarios, including but not limited to PT heterodyne gas detection, spatial-modes-based physical field sensing, and interplanetary coherent communications.

## 2 Theoretical analysis and method

### 2.1 Acoustic-induced mode-dependent low-frequency shifter

**Figure 1:**
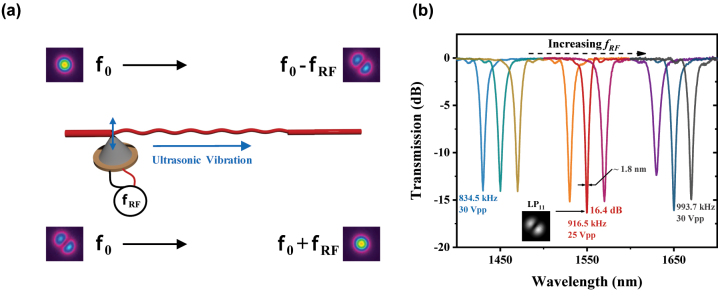
Principled diagram and transmission spectrum of AIFG. (a) Characteristics of the transverse modes conversion and frequency shift, (b) transmission spectrum of AIFG. AIFG, acoustic-induced fiber grating; *f*_0_, frequency of incident light; and *f*_*RF*_, frequency of applied RF signal.

As the key component of all-fiber AOFS, the AIFG formed by the acousto-optic interaction (AOI) acts as an adjustable dynamic fiber grating [21]. It not only realizes an efficient conversion between the adjacent linear polarization modes: LP_o,n_ and LP_l,n_ (LP_01_ and LP_11_ here), but also leads to an optical mode-dependent frequency shift [19–21], as illustrated in Figure 1a. When the ultrasonic wave generated by the piezoelectric transducer (PZT) is transmitted to the unjacketed FMF through the mechanical connection between the aluminum cone and the FMF, the axial micro perturbation of the core will accordingly modulate the refractive index, leading to the index-modulation characteristic of the dynamic long-period grating (DLPG) [22]. According to the acoustic dispersion theory, the period of the generated DLPG can be expressed as: $\Lambda ={\left(\pi RC_{\text{ext}}/f_{RF}\right)}^{1/2}$ , where *C*_ext_ and *f*_*RF*_ refer to the velocity and frequency of the ultrasonic wave propagating in silica, *R* is the radius of the fiber [23]. The mode conversion between the LP_01_ and LP_11_ occurs when the phase-matching condition is satisfied: $L_{B}=\lambda_{r}/\left(n_{01}-n_{11}\right)=\Lambda$ , in which *L*_*B*_ refers to the beat length between the two modes, *λ*_*r*_ denotes the resonant wavelength, *n*_01_ and *n*_11_ represent the effective refractive indices of LP_01_ and LP_11_, respectively [14, 17, 24]. Additionally, the conversion efficiency as well as the resonant wavelength can be dynamically adjusted by varying the frequency and amplitude of the applied RF signal, which can be interpreted by the following form [18, 25, 26]:

(1) $$\begin{array}{ll} \kappa_{01-11} & =\frac{\pi }{\lambda_{r}}\sqrt{\frac{\varepsilon_{0}}{\mu_{0}}}n_{0}\iint E_{01}\left(x,y\right)\cdot E_{11}\left(x,y\right)\\ & \;\cdot \left[n_{0}\left(1+\chi \right)K^{2}A_{0}x\right]dxdy \end{array}$$

where *E*_01_(*x*,*y*) and *E*_11_(*x*,*y*) refer to the cross-sectional electric-field distribution of the transverse modes LP_01_ and LP_11_. And *n*_0_, *χ*, *A*_0,_ and *K* represent the refractive index of the FMF core, the elasto-optic coefficient of silica, and the amplitude and wavevector of the ultrasonic wave, respectively. When the broadband source is guided through the AIFG with a segment of the SMF to cut off the converted LP_11_ mode, the transmission spectrum of the AIFG with wavelength-tuning characteristics [27] can be obtained by adjusting the *f*_*RF*_ and *A*_0_, as shown in Figure 1b. It implies that mode-conversion efficiencies surpassing 10 dB can be attained within a dynamic tuning range that spans nearly 250 nm. In particular, an efficiency of 16.4 dB (>97.5 %) and a 3-dB bandwidth of 1.8 nm can be achieved at the wavelength of 1550 nm, when a RF signal with *f*_*RF*_ of 916.5 kHz and *A*_0_ of 25 Vpp is employed.

Theoretically, it has been revealed that the acoustic-induced in-fiber perturbation will contribute to the permittivity change, which is composed of the physical points’ geometrical displacement and fiber properties change due to the photoelastic effect [18, 21, 28]. This process will lead to a mode-dependent optical frequency shift, which will be superimposed on the converted mode. For instance, in the case that the incident light is lower-order mode and the photon-phonon pair is co-propagated, the hybrid modes (converted mode and non-converted mode, LP_11_ and LP_01_ here) from the AOI can be expressed as the following form [21]:

(2) $$\Psi \left(z\right)=\left[c_{01}^{\sigma }\left(LP_{01}^{\sigma }\right)+c_{11}^{\sigma }\left(LP_{11}^{\sigma }\right){\text{e}}^{\text{i}\left(2\pi f_{RF}t-Kz\right)}\right]e^{-i2\pi f_{0}t}$$

here, the first and second terms represent the incident mode (LP_01_) and the converted mode (LP_11_), $c_{01}^{\sigma }$ and $c_{11}^{\sigma }$ are their proportions. The *f*_0_ and *f*_*RF*_ are the frequencies of the incident mode and applied RF signal, respectively. As is seen, the converted mode LP_11_ is superimposed with a frequency downshift: *f*_0_ − *f*_*RF*_. And so on, four frequency-shift cases dependent on the two incident modes as well as the two photon-phonon correlative-propagating directions can be further summarized in Table 1. Note that, the frequency shifts with same magnitudes but opposite directions will be obtained by directly exchanging the incident mode and converted mode, which also shows the non-reciprocity resulting from the AOI. Additionally, from the quantum mechanical point of view, the photon-phonon scattering within the AOI also satisfies the conservation of energy and momentum: (*f*′, *K*′) = (*f*, *K*) + (*f*_*s*_, *K*_*s*_), where (*f*′,*K*′), (*f*,*K*), and (*f*_*s*_,*K*_*s*_) denote to the frequency and wavevector of the converted photon, incident photon, and propagating phonon, respectively [17, 20, 21]. Among them, the superposition of frequencies: *f*′= *f* + *f*_*s*_ and *f*_*s*_ = ± *f*_*RF*_ are the physics of frequency shift.

**Table 1:** Directionality of the acoustic-induced mode-dependent frequency shift.

Transverse mode conversion	Photon-phonon correlative direction	Frequency of the converted mode
LP_01_ to LP_11_	Co-propagating: → →	Downshift: *f*_0_ − *f*_*RF*_
	Counter-propagating: → ←	Upshift: *f*_0_ *+ f*_*RF*_
LP_11_ to LP_01_	Co-propagating: → →	Upshift: *f*_0_ *+ f*_*RF*_
	Counter-propagating: → ←	Downshift: *f*_0_ − *f*_*RF*_

*f*_0_, optical frequency of the incident mode; *f*_*RF*_, frequency of the applied RF signal.

**Figure 2:**
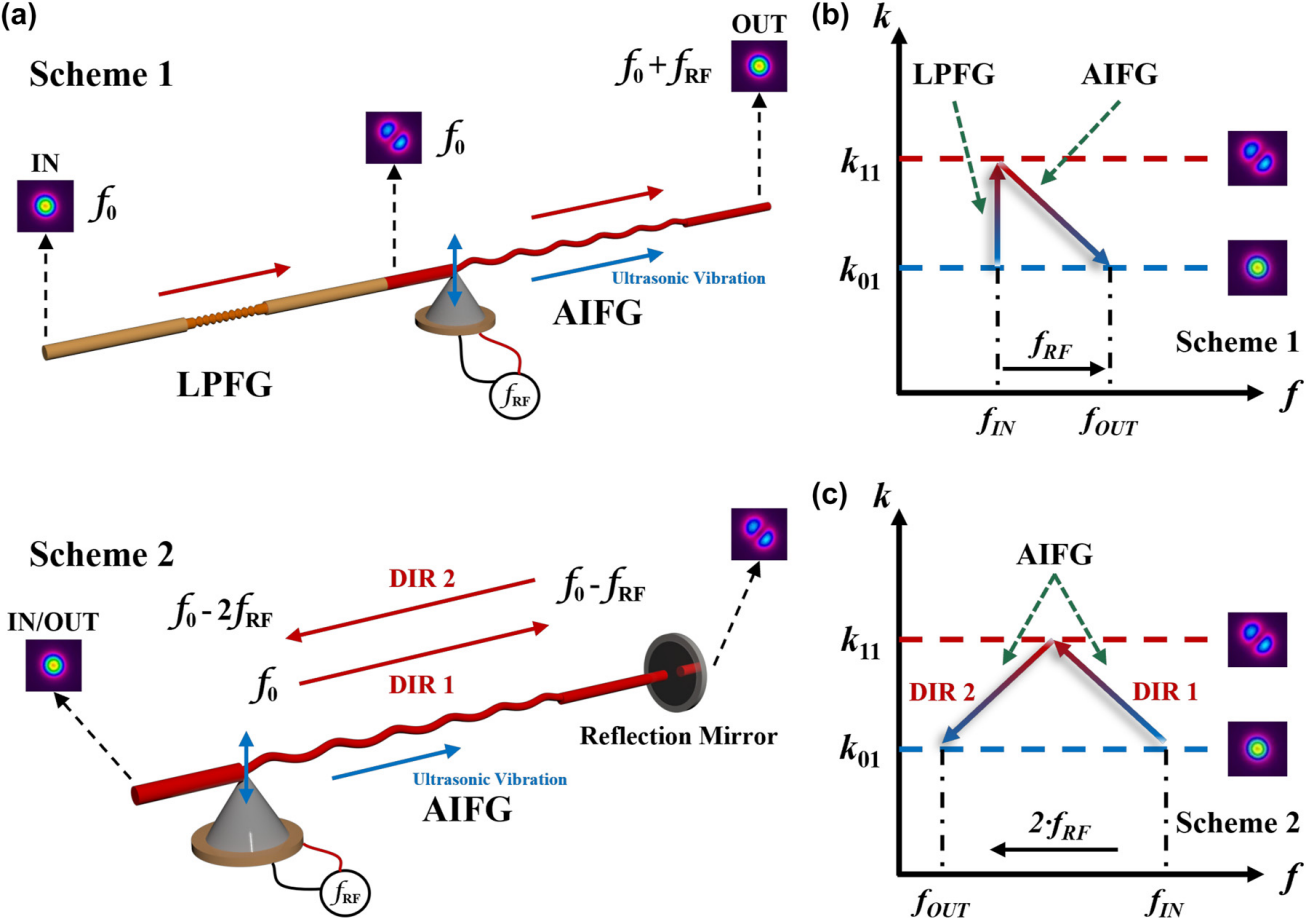
The structure design of the AOFSs. (a) Two schemes of all-fiber AOFSs based on acoustic-induced cyclic mode conversion and their mode-dependent optical frequency shift, (b) frequency shift direction of Scheme 1, (c) frequency shift direction of Scheme 2. AOFS, acousto-optic frequency shifter; DIR, direction of propagating light; LPFG, long-period fiber grating; *f*_0_, frequency of incident light; *f*_*RF*_, frequency of applied RF signal.

Based on the aforementioned explanation of the acoustic-induced cyclic conversion of transverse modes and the mode-dependent optical frequency shift, two schemes are proposed including the AIFG with the LPFG or cascading the AIFGs in counter-propagation through the reflection mirror. The structures of proposed AOFS schemes, as well as their corresponding frequency shift directions, are depicted in Figure 2, individually. Scheme 1 in Figure 2a consists of two mode-conversion devices: LPFG and AIFG. The LPFG written in a two-mode fiber (*r*_core_/*r*_cladding_: 9/125 μm, *n*_core_/*n*_cladding_: 1.476/1.468) with a period of 342 μm and period number of 14 converts the LP_01_ to LP_11_, detailed in the work [29]. And the AIFG formed in a four-mode fiber (*r*_core_/*r*_cladding_: 18.5/125 μm, *n*_core_/*n*_cladding_: 1.467/1.462) converts the LP_11_ back to LP_01_ and produces an optical frequency upshift (equals to the frequency of the RF signal), as shown in Figure 2b. Meanwhile, the reflection path of Scheme 2 employs a cascading of two AOI regions that can realize the cyclic conversion of transverse modes and an optical frequency downshift (equals to twice of the frequency of the RF signal) in one single AIFG device, as shown in Figure 2c. The LP_01_ mode is converted to LP_11_ mode in direction 1 (photon-phonon co-propagating) and then converted back to LP_01_ mode in direction 2 (photon-phonon counter-propagating). The directions of the optical frequency shifts of these two schemes at each stage are given in Table 1 above.

**Figure 3:**
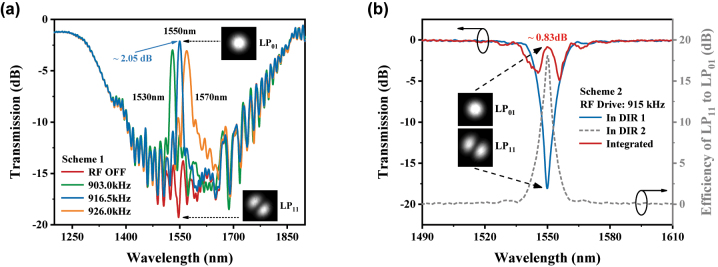
Characterization of two AOFS schemes. (a) Transmission spectrum of Scheme 1 and (b) transmission spectrum of Scheme 2.

These two AOFS schemes are experimentally realized and the transmission spectra are exhibited in Figure 3. In Scheme 1, the red line in Figure 3a represents the case without applying the RF signal, which shows the original transmission characteristic of single LPFG as a matter of fact. Solid lines in green, blue, and orange indicate the different resonant wavelengths corresponding to the applied RF signal with different frequencies. Particularly, when the RF signal is 916.5 kHz, an AOFS providing a frequency upshift of 916.5 kHz can be obtained at the operating wavelength of 1550 nm. The insertion loss of 2.05 dB for Scheme 1 is mainly caused by the incomplete mode conversion and the mismatched mode field between two different FMFs for AIFG and LPFG. In Scheme 2, the optical reflection path is implemented by a customized jumper with an end-face metal coating (reflectivity > 99.5 %, represented by the ‘reflection mirror’ in the figure). As shown in Figure 3b, the red solid line shows the integrated transmission spectrum when an RF signal of 915 kHz is applied (the gray dotted line represents the transmission spectrum of direction 2, which cannot be directly measured). The tiny dual-deeps around 1550 nm in red solid line are caused by the symmetric transmission characteristic of the AIFG in both directions among the reflection path. With the cancellation of the LPFG within the scheme, higher purity of cyclic conversion can be obtained without the mode-field mismatch between LPFG and AIFG. An insertion loss lower than 0.83 dB can be obtained as well as the same wavelength tuning performance compared with Scheme 1.

**Figure 4:**
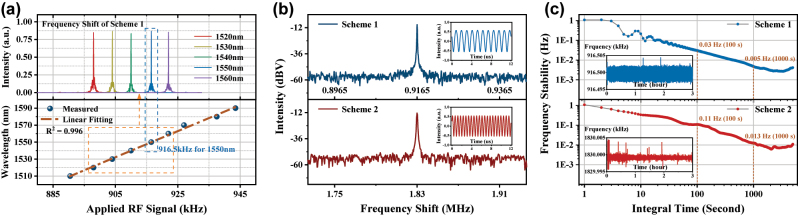
Characterization of two AOFS schemes. (a) Tuning frequency spectrum of Scheme 1 and the proportional relationship between the RF signal and the resonant wavelength, (b) the frequency spectra and time-varying waveforms of two schemes, and (c) stability analysis of the two AOFSs through Allan Variance.

Furthermore, as shown in Figure 4, we test the tuning performances, frequency shift intensities and robustness of both schemes via placing them on the one arm of a heterodyne interferometer. The directly proportional relationship between the frequency of applied RF signal and resonant wavelengths of Scheme 1 is shown on the bottom half of Figure 4a. The continuous tuning region from 1510 nm to 1590 nm can be obtained by adjusting the RF signal from 890.5 kHz to 943.5 kHz (6.6 kHz/10 nm). Meanwhile, the frequency-shift spectra corresponding to 5 equally spaced wavelengths from 1520 nm to 1560 nm are shown on the top half of Figure 4a (Light source: *LUNA Phoenix 1400*, Photodetector: *HAMAMATSU InGaAs PIN*). Figure 4b represents the frequency spectra of these two AOFS schemes in blue and red lines: center frequency of 916.5 kHz and 1.83 MHz (twice of 915 kHz), respectively. For both schemes, the shift components with sideband rejection ratios over 40 dB can be simultaneously obtained. The blue and red insets in Figure 4b are their waveforms in time domain. Since the AIFGs in both schemes use the same FMF, the negligible difference of the optical frequency shifts between the two schemes (916.5 kHz for Scheme 1, 915 kHz for Scheme 2, at 1550 nm) within a single AOI region is only the small deviation generated during the fabrication process. As shown in Figure 4c, the frequency-shift stabilities of the two AOFSs within 3 h are analyzed using Allan Variance. When the integration time is set to 100 s, the standard deviations of the frequency shift are 0.03 Hz and 0.11 Hz, respectively, which include errors inherent to the signal generation equipment itself. Both AOFSs are encapsulated in customized boxes for long-term stability and integration with other devices.

### 2.2 Microfiber evanescent-field photothermal effect

**Figure 5:**
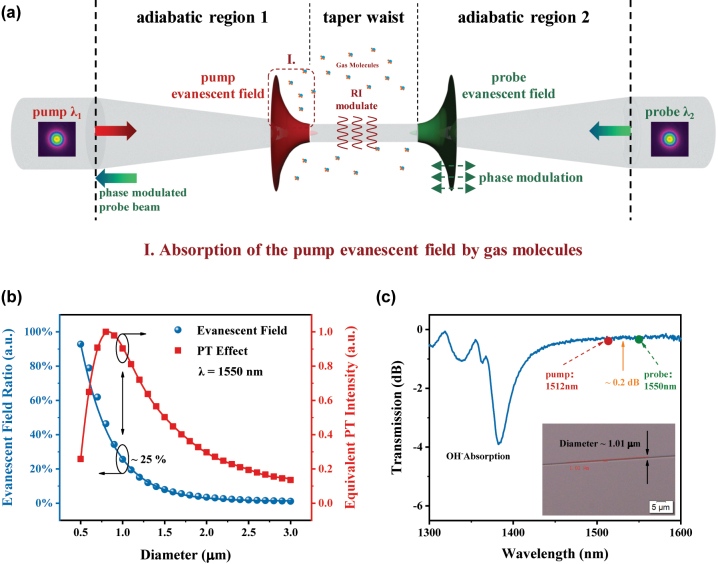
Microfiber-based photothermal effect. (a) Principle of the microfiber evanescent-field PT gas detection, (b) simulation of the evanescent field intensity and the normalized equivalent PT effect intensity, and (c) transmission spectrum of fabricated microfiber with a diameter approximately to 1 μm. Inset in (c): microfiber guiding a supercontinuum source and its electron microscope image.

As depicted in Figure 5a, a microfiber acts as the sensing medium for PT gas detection. It is adiabatically tapered to have the gently transitioning taper angle as well as the nano-scale waist diameter, which leads to evanescent field with great intensity and transmission characteristics of nearly lossless and no modal interference [30–34]. The absorption of the modulated pump-light evanescent field by gas molecules acts as the heat source, which can cause the temperature change of the medium (microfiber and near-field gas) via thermal conduction (shown as **I** in Figure 5a). They jointly modulate the refractive index (RI) of the local sensing medium [9, 10]. Hence, the mode effective RI of the probe light would be modulated accordingly if the pump-probe light pair are collinear-propagating [7], and can be expressed as: $\Delta n_{\text{probe}}=T_{f\left(a\right)}\left(r,\theta ,t\right)\Delta \varepsilon_{f\left(a\right)}$ . Here, *T*_*f*(*a*)_(*r*,*θ*,*t*) and *ε*_*f*(*a*)_ are the temperature modulations and the thermal-optical coefficients (TOCs) of the microfiber (silica) and surrounding air, respectively. Meanwhile, the effective sensing length (approximate to the ‘taper waist’ in Figure 5a) causes the accumulative effect of the phase modulation of the probe light. Combining the radial effective RI modulation of the probe light and the axial accumulative effect, the probe light phase modulation *Δθ*_probe_(*t*) caused by the PT effect could be summarized as the following [9, 12, 35]:

(3) $$\begin{array}{ll} \Delta \theta_{\text{probe}}\left(t\right) & \propto \frac{2\pi }{\lambda_{\text{probe}}}k\alpha \left(\lambda_{\text{pump}}\right)C_{\text{gas}}L_{\text{eff}}D_{\text{pump}}\left(r,\theta \right)I_{\text{ave}}g_{\text{pump}}\left(t\right) \end{array}$$

which is relevant to the gas concentration *C*_gas_, the effective sensing length *L*_eff_ and the pump average power *I*_ave_. Here, *α*(*λ*_pump_), *D*_pump_(*r*,*θ*), and *g*_pump_(*t*) are the absorption coefficient (as a function of pump wavelength), the normalized distribution of pump light which is composed of evanescent field and in-fiber field, and the pump modulation waveform, respectively. The parameter *k* is related to the pump modulation frequency. In particular, *D*_pump_(*r*,*θ*), the distribution of the evanescent field, is assumed to be a Gaussian function [36]:

(4) $$D_{\text{pump}}\left(r\right)=\frac{2}{\pi \tau_{\text{pump}}^{2}}\exp\left(-\frac{2r^{2}}{\tau_{\text{pump}}^{2}}\right)\propto \frac{1}{\tau_{\text{pump}}^{2}}$$

where, *τ*_pump_ = *r*_fiber_ + *d*_*p*_, is the effective mode field radius of the pump light propagating through the microfiber. The *r*_fiber_ and *d*_*p*_ refer to the radius of the microfiber and penetration depth of the evanescent field, respectively. Here, the *d*_*p*_ can be solved as 350 nm when the wavelength is about 1.5 μm [37], then *τ*_pump_ is 850 nm if the microfiber diameter is 500 nm. Compared with the typical beam radius that 5 μm for HCF or 50 μm for free-space light, microfiber boosts the power density in two to four orders of magnitudes. In addition, since the absolute value of the TOC of silica is approximately 10 times that of air (*ε*_air_ = −0.9 × 10^−6^/K, *ε*_silica_ = 8.1–9.7 × 10^−6^/K [38, 39]), the PT effect could be further enhanced by microfiber. However, it is a remarkable fact that the opposite signs among the TOCs of the air and silica also lead to the optimization of the microfiber diameters [9]. Whereupon, compared with the HCF and free-space light, the microfiber evanescent field can enhance the PT phase modulation of the probe light under the same pump average power *I*_ave_ and effective sensing length *L*_eff_.

The evanescent field ratio and equivalent PT effect intensity are simulated under a microfiber diameter from 500 nm to 3000 nm, and the step size is set to 100 nm (via *COMSOL Multiphysics*). The microfiber model is assumed to be an ultra-slim silica wire with a RI consistent with the cladding RI of standard SMF (*Conning SMF-28e*). Via solving the surface integral of power density among the microfiber and air in cross-section, the tendency that the evanescent field ratio increases exponentially with the decrease of the microfiber dimension can be obtained, and shown as the blue line in Figure 5b. Moreover, according to the penetration depths under different microfiber diameters as well as the TOCs of the air and silica, the normalized equivalent PT effect intensity is estimated as the red line in Figure 5b. The simulation result shows that the maximum could be obtained when the diameter of the microfiber is 800 nm.

Here, we fabricate the microfiber with a diameter of about 1 μm, which is adiabatically tapered from the SMF. According to the simulation results above, approximately 25 % of the pump light propagates on the fiber surface in the form of evanescent field. The lengths of the slow-changing taper region (‘adiabatic taper region 1 and 2’ in Figure 5a) and uniform taper waist (‘taper waist’ in Figure 5a) are 34 mm and 4 mm, fulfilling the adiabatic criteria [40]. The characterization by guiding a supercontinuum source is shown in Figure 5c as well as the inset shows the microscope image (via *Olympus BX53M @50x*). Since the surface smoothness of the taper waist supports the stable transmission without modal interference, the insertion loss at the telecommunication band is 0.05 dB/mm, and the great depression near 1385 nm is resulting from the absorption by OH^−^ inside the fiber. Then, the UV glue is used to fix the microfiber sample on a U-shaped glass groove, which is settled on a customized slide. The encapsulated samples are then gently placed in a Teflon-coated glass jar as a microfiber gas absorption cell for the subsequent experiment.

## 3 Experimental setup, results, and discussion

### 3.1 Experimental setup

**Figure 6:**
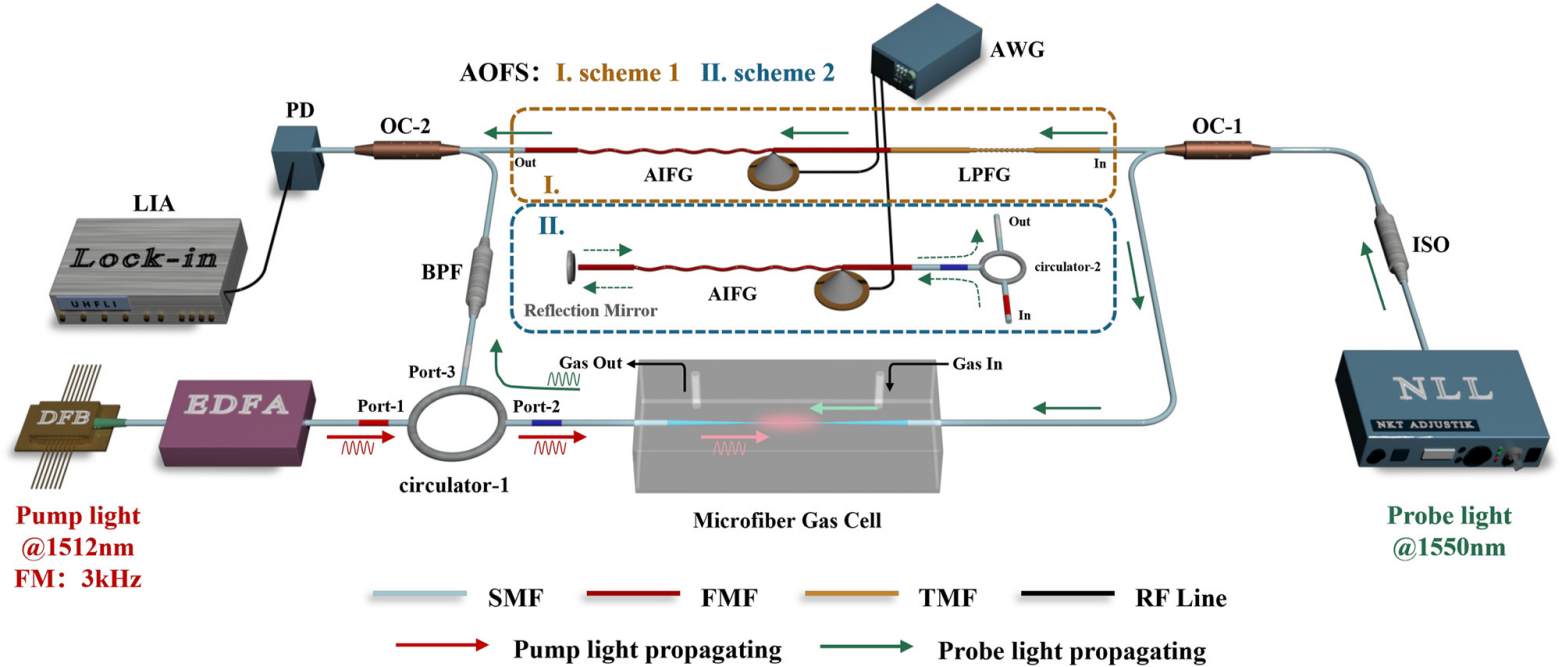
Schematic diagram of experimental setup for the evanescent-field PT heterodyne gas detection using acoustic-induced mode-dependent frequency shifter. DFB, distributed feedback laser (center wavelength: 1512 nm); NLL, narrow-linewidth laser (center wavelength: 1550 nm); EDFA, erbium-doped optical fiber amplifier; PD, photodetector; LIA, lock-in amplifier; OC, optical coupler; AWG, any waveform generator; BPF, band-pass filter (@1550 ± 1.5 nm); ISO, optical isolator (@1550 ± 30 nm); AIFG, acoustic-induced fiber grating; LPFG, long-period fiber grating; AOFS, acousto-optic frequency shifter; SMF, single-mode fiber; FMF, few-mode fiber; TMF, two-mode fiber; RF line, radio frequency line; and FM, frequency modulation.

The experimental setup for evanescent-field PT heterodyne gas detection using acoustic-induced mode-dependent frequency shift is shown in Figure 6.

A counter-propagating pump-probe configuration is set for a more compact structure with fewer devices [12]. A DFB laser (*LD-PD INC PL-DFB @1512 nm*) and a narrow linewidth laser (*NKT Photonics E15 @1550 nm*) are configured as the pump light and probe light. The DFB laser is driven by a current signal composed of a slow-varying sawtooth wave (200 mHz) for wavelength tuning and a rapid-varying sinusoidal wave (3 kHz) for wavelength modulation. The scanning wavelength of pump light can be tuned across the ammonia absorption line (1512.24 nm) along with a superimposed wavelength modulation (*f*_*m*_ = 3 kHz). The pump light is amplified to about 40 mW via an EDFA (*Shanghai Teraband 1550 OA*) and then injected into the homemade microfiber gas cell to excite the PT effect. Meanwhile, the probe light is divided into two beams by the optical coupler (OC-1). One serves as the sensing beam which is injected into the gas cell for obtaining the cumulative PT phase modulation, and another acts as the reference beam which is guided to the proposed AOFS scheme to produce a low-frequency shift of sub-MHz for heterodyne detection. Due to the acoustic-induced mode-dependent frequency shifts of AOFSs, the low-frequency shift that 916.5 kHz of Scheme 1 and 1.83 MHz of Scheme 2 can be achieved on the reference arm, respectively. Then, the probe lights through the reference arm and sensing arm are mixed by the OC-2. The photomixing beam is superimposed onto a PD, which is used to record the heterodyne beat signal. Subsequently, the phase of the beat signal will be demodulated via the LIA (*Zurich Instruments: MFLI@5MHz*). It is noteworthy to note that, the powers of the probe lights in two arms should keep roughly the same in order to obtain good stability of the beat signal [14, 41]. Two adjustable attenuating flanges are utilized at both outputs of the OC-1 to ensure the balance of the powers at the inputs of the OC-2.

**Figure 7:**
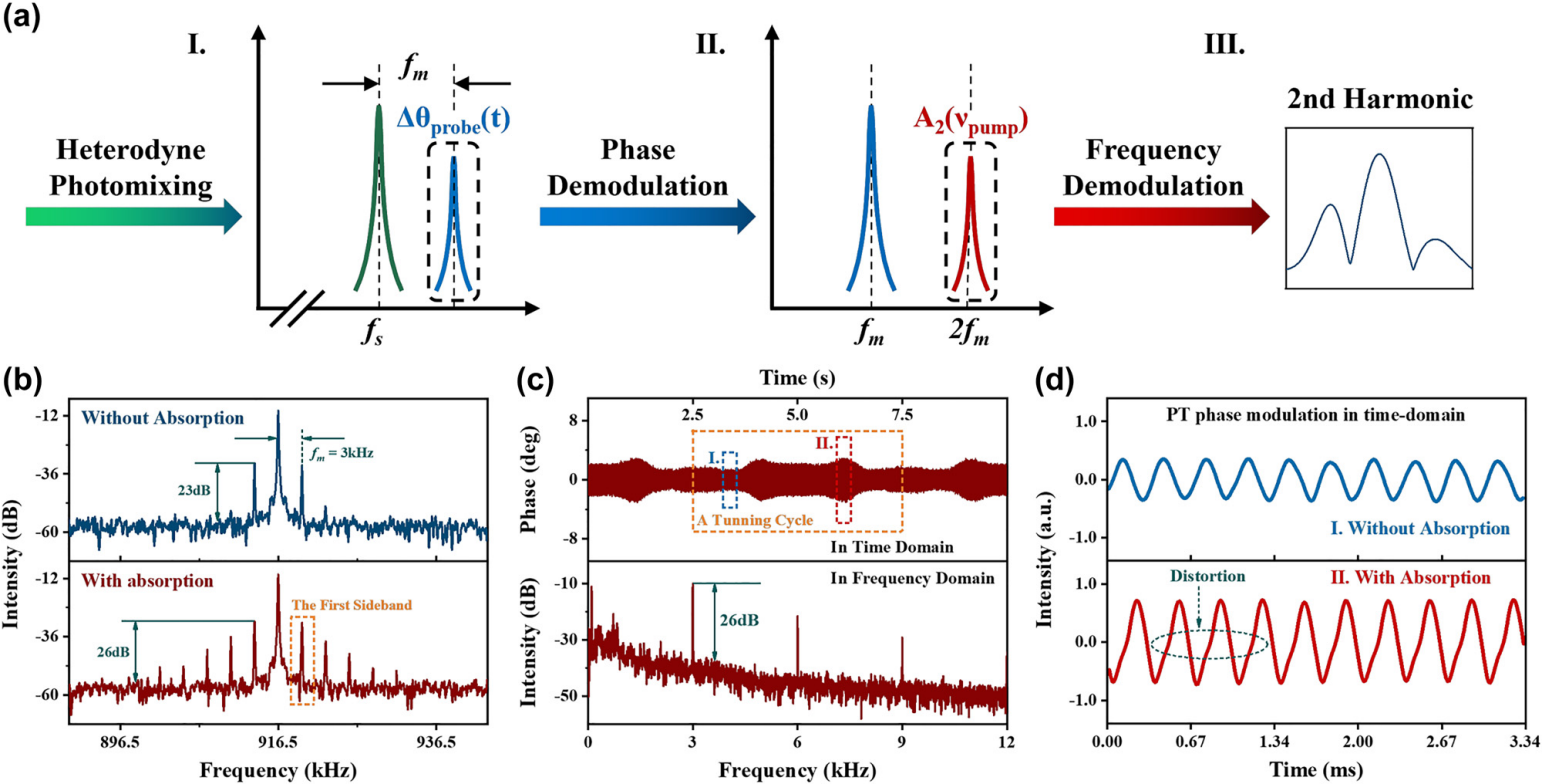
Heterodyne beat signal carries PT phase modulation and its demodulation. (a) Flow diagram of the heterodyne detection and its two-step demodulation, (b) beat signals in the frequency domain (blue: without ammonia absorption, red: with ammonia absorption), (c) frequency spectrum and time-domain waveform of the beat signal when the gas absorption occurs, and (d) comparison of the PT phase modulation waveforms when the scanning wavelength of the pump light is at (red) and away (blue) from the absorption line of ammonia. *f*_*s*_, heterodyne frequency of the beat signal; and *f*_*m*_, modulation frequency of the pump light.

Figure 7a shows the flow diagram retrieving the gas information via demodulating the phase of the probe light. The output photomixing signal from the interferometer consists of sum- and difference-frequency components. Since the PD only responds to the difference-frequency component, the beat signal may be simply expressed as follows [8]:

(5) $$E_{\text{beat}}\left(t\right)=A_{\text{beat}}\cdot \cos\left[2\pi f_{s}t+\Delta \theta_{\text{probe}}\left(t\right)\right]$$

where *A*_beat_ and *f*_*s*_ = *f*_*RF*_ are the amplitude and central frequency of the heterodyne beat signal. The probe light PT phase modulation *Δθ*_probe_(*t*) can be obtained by demodulating the phase of the beat signal (shown as **I** in Figure 7a). The absorption coefficient within the pump-light tuning cycle is nonlinear: $\alpha \left(\lambda_{\text{pump}}\right)=\alpha \left[\lambda_{\text{pump0}}\cdot \left(1+g_{\text{pump}}\left(t\right)\right)\right]$ , where $g_{\text{pump}}\left(t\right)=u\left(t\right)+\eta \cos\left(2\pi f_{m}t\right)$ is the modulation waveform for the pump light, *λ*_pump0_ and *η* are the central wavelength of pump light and the modulation depth, *u*(*t*) and cos(*2πf*_*m*_ *t*) refer to the sawtooth signal and sinusoidal signal, respectively. If the absorption of the pump light by the gas molecules occurs, the phase of the probe light will become distorted [12, 42, 43], and some new frequency components would be generated (shown as **II** in Figure 7a). Hence, by applying Fourier expansion, the n-order harmonic signal linearly proportional to the *C*_gas_, *L*_eff_, and *I*_ave_, can be expressed as:

(6) $$\begin{array}{ll} A_{n}\left(\lambda_{\text{pump}}\right) & \propto kC_{\text{gas}}L_{\text{eff}}D_{\text{pump}}\left(r,\theta \right)I_{\text{ave}}\\ & \;\times \frac{d^{n}\alpha \left(\lambda_{\text{pump}}\right)}{d\lambda^{n}}{|}_{\lambda =\lambda_{\text{pump}}} \end{array}$$

As shown in the top half of Figure 7b, the heterodyne central frequency when applying Scheme 1 is 916.5 kHz. When the pump light is on and no gas has been loaded, two small peaks with intensities of 23 dB will appear by the sides of the heterodyne center with an interval of *f*_*m*_, which represent the initial PT phase modulation caused by the pump light itself. In particular, the optimizable parameter of *f*_*m*_ will be detailed later. However, as shown in the bottom half of Figure 7b, when the ammonia is loaded into the microfiber cell and the pump wavelength tunes onto the ammonia absorption line, the beat signal carries some additional evenly spaced sidebands. The stronger first sideband with an intensity of 26 dB and those sidebands separated by *f*_*m*_ represent the harmonics relevant to the additional PT effect resulting from the gas absorption of the pump evanescent field. When the phase demodulation is performed at the heterodyne center frequency (916.5 kHz) and the cut-off bandwidth of the low-pass filter is set greater than 3 kHz, the first sideband of the beat signal can be obtained solely and its spectrum and time-domain waveform are shown in Figure 7c. Region **II** in the top half of Figure 7c corresponds to the waveform in the bottom half of Figure 7d. At this moment, the distorted PT phase modulation waveform with higher amplitude is the outward manifestation of the nonlinear process within the gas absorption and implies that it can be demodulated into harmonics. We choose the second harmonic as the detection signal, whose amplitude is linearly proportional to the *C*_gas_, as explained in Eq. (6). Then it can be obtained by demodulating the PT phase modulation signal when the frequency of the reference signal offered by the local oscillator is set to *2f*_*m*_ in the LIA.

### 3.2 Experimental results and discussion

**Figure 8:**
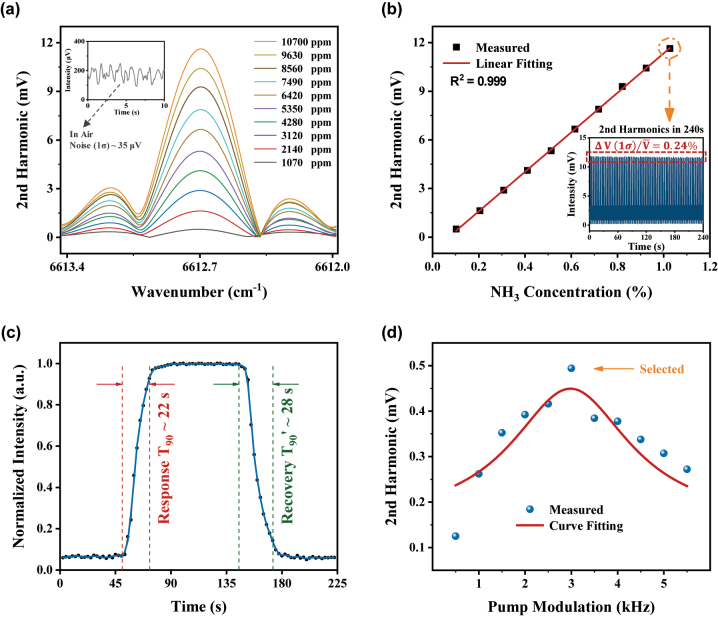
Experimental results of NH_3_ detection. (a) Second harmonics measured under an ammonia concentration gradient from approximately 0.1 %–1 %, (b) linear fitting for the second harmonics, (c) characteristic of fast response and recovery, and (d) second harmonic varies with the pump light modulation *f*_*m*_. Inset of (b): Stability measurement within 240 s (48 pump tunning cycles).

1512.24 nm (wavenumber: 6612.7 cm^−1^, absorption line intensity: 2.26 × 10^−21^ cm^−1^/(molec·cm^−2^) [44]) is chosen as the central absorption wavelength for ammonia. The ammonia with a concentration gradient from approximately 0.1 %–1 % is loaded into the microfiber gas cell in sequence. As shown in Figure 8a, the second harmonics under different concentrations are obtained via demodulating the PT phase modulation signal at 6 kHz (twice of the *f*_*m*_ = 3 kHz). Via the *C*_gas_ of 10,700 ppm, the second harmonic of 11.63 mV and the 1*σ* noise (in air) of 35 μV, the equivalent detection limit (1*σ*) could be estimated as 32 ppm. Subsequently, as shown in Figure 8b, the fitting result based on the second harmonics’ amplitudes indicates a well-linear relationship with the concentration. Furthermore, the second harmonics within 48 pump-light tuning cycles (tuning frequency: 200 mHz) are successively sampled to estimate the consistency, shown in the inset of Figure 8b. The standard deviation (1*σ*) is calculated as 28 μV, resulting in a signal fluctuation of 0.24 %, which signifies an excellent stability of the whole system. The response characteristic of the system is also obtained by recording the second harmonics’ amplitudes with an interval of 2.5 s. The normalized amplitudes and their evolving curve show that the response time *T*_90_ (which takes to reach 90 % of the peak) is about 22 s and the recovery time is about 28 s, illustrated in Figure 8c. It is worth noting that the response time is constrained by the volume of the gas chamber (around 300 mL) and the pumping speed (around 300 mL/min) of the gas. Compared with the HCF, the fast response recovery is the result of the direct contact between the gas molecules and the evanescent field. Meanwhile, since there is no need for a complex upfront fabrication process like local micro-operation, the microfiber gas cell can easily scale up to large-scale arrays for flexible deployment, which significantly expands its practical potential.

In addition, as explained in Eq. (6), the greatest SNR could be accordingly achieved when an optimal value of the pump-light modulation parameter is set. As shown in Figure 8d, the optimal modulation parameter of 3 kHz is obtained by comparing the second harmonics’ amplitudes under different modulation frequencies when the ammonia concentration is 0.1 %. It is worth noting that, with the increase of the length difference between the two arms of the heterodyne interferometer, the phase noise caused by the probe light itself and ambient disturbance will accumulate rapidly. Whereupon, the detection sensitivity can be effectively enhanced by precisely controlling the optical path lengths of the probe arm and reference arm [8]. In our experiment, the optical path-length differential between the two arms is controlled and estimated to be 100-mm level. We believe that an equivalent detection limit of sub-ppm level may be obtained by precisely adjusting the arm length difference within 10 mm or less.

**Figure 9:**
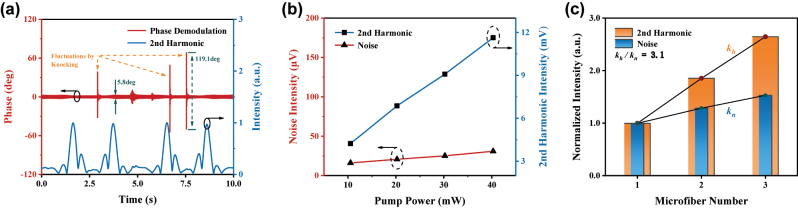
Intrinsic properties of the microfiber-based photothermal system. (a) Immunity to ambient noise, (b) sensitivity enhancement by boosting the pump power, and (c) sensitivity enhancement by applying the microfiber array. *K*_*h*(*n*)_: normalized growth rates of the second harmonic and noise, respectively.

Furthermore, as a promising second-order sensing method, the detection signal generation depends on not only the analytical gas sample but also the laser optics itself [45]. Some other interesting features of this evanescent-field PT gas detection system like immunity to ambient noise, PT enhancement from the pump light boosting, and application of microfiber array, are also investigated shown in Figure 9.

As shown in Figure 9a, the red curve represents the PT phase modulation signal and the blue curve shows the second harmonic. When the gas detection platform is subjected to physical disturbance like violent knocking, the ambient vibration is conducted to the microfiber, and great phase fluctuations of the probe light will be excited by the instantaneous perturbation of the microfiber. This is also the principle of the laser-based micro-vibration monitor and sound listener [14, 17, 46], [47], [48]. However, unlike the nonlinear absorption by gas, the noise caused by ambient vibration is limited to alter the amplitude of probe light phase modulation but almost does not distort it, which means that there will be few new frequency components to be generated. Therefore, the demodulated second harmonic remains stable, which reflects the stability of this sensing system.

As explained in Eq. (6), the amplitude of the second harmonic is also proportional to the pump light power. This is due to the fact that higher pump power leads to stronger PT effect, causing greater distortion of the probe-light phase modulation. Figure 9b displays a comparison of the corresponding second harmonic amplitudes and noise levels for increasing pump power, under an ammonia concentration of about 1 %. It is clear that the growth rate of noise is less than that of the second harmonic, and the ratio of the two increases from 264 to 375 when the pump power is set from 10.53 mW to 40.27 mW, which shows a good linear-growth trend. Ideally, noise does not increase with the pump power increasing, which results in a multiple heightening of SNR. Therefore, this feature indicates that the sensing sensitivity can be enhanced via the application of a well-performance EDFA with a high gain coefficient.

Benefiting from the compact and flexible configuration, the use of microfiber leads to not only the advantages of fast response recovery and PT enhancement. The possibility of the application of the microfiber array is also simply demonstrated, expressed in Figure 9c. A larger effective sensing length can be achieved, improving the sensing sensitivity to some extent. We fabricate a cascading-type multi-microfiber array for testing the enhancement, and each tapered microfiber has an approximate waist diameter. For comparison, the second harmonics and noises are recorded under a microfiber number of 1, 2, and 3, they are then, respectively, normalized in the case of single microfiber. With the increase of the microfiber, the normalized ratio between the growth rates that the second harmonic and noise could be expressed as: *k*_*h*_ */k*_*n*_ = 3.1. This result shows that further large-scale microfiber arrays can be flexibly applied to enhance the detection sensitivity.

The gas detection performances obtained by using the proposed two AOFS schemes are also listed in Table 2. As expressed in Section 2.1. According to the amplitudes of the second harmonic and noise under the *C*_gas_ of about 1 %, both AOFS schemes can obtain an equivalent detection limit (1*σ*) of about 20–30 ppm. Comparative results show that both schemes lead to excellent low-frequency shift performances and can be effectively used in heterodyne detection systems as well as related fields.

**Table 2:** Performance comparison using the proposed two AOFS schemes under 1 % ammonia.

AOFS	^a^Frequency shift (MHz)	^a^Frequency stability (Hz)	Noise (μV)	2nd harmonic (mV)	SNR	Detection limit (ppm)
Scheme 1	0.9	0.03 (integration: 100 s)	35	11.63	332	32
Scheme 2	1.83	0.11 (integration: 100 s)	36	18.23	506	21

AOFS, acousto-optic low-frequency shifter; SNR, signal-to-noise ratio, ^a^self-characterization.

We finally compare the configurations and parameters of different PT gas sensors, summarized in Table 3. In [5], a PZT-based servo control loop is needed for achieving the quadrature point, which increases the instability and complexity. Compared with the homodyne method, the application of heterodyne interferometry makes the configuration much simpler. However, as stated in [8, 49], two cascaded LIAs or an ultrahigh-frequency-capable LIA are used for the demodulation of the heterodyne beat signal with an ultrahigh central frequency of hundreds MHz, which makes the demodulation process cumbersome and costly. Meanwhile, the multi-pass gas cell like [50] requires a complex mechanical structure, while the recent PT gas sensors using HCF result in an ultrahigh response time of hours because the HCF should be filled by analyte in advance, as depicted in [5, 51]. Compared with the configurations of conventional PT gas sensors, the acoustic-induced mode-dependent low-frequency shifters that we propose can efficiently implement all-fiber integration and reduce the cost of demodulation, replacing the AOM effectively. Meanwhile, the adiabatic microfiber array also leads to excellent performance, which is expected to be the substitution of the multi-pass cell. Their features give rise to promising prospects in PT gas detection.

**Table 3:** Performances and configurations comparison of different PT gas sensors.

Gas	Pump wavelength	Heterodyne	Gas cell	Effective sensing	Full-response	Equivalent detection
	(μm)	frequency (MHz)		length (cm)	time (s)	limit (ppm)
C_2_H_2_ [5]	1.53	N/A (homodyne method)	HC-PBF	1000	7200 (for filling)	0.002
NO [50]	5.25	40	Herriot multi-pass	364	Not stated	10 (integration: 100s)
N_2_O [8]	3.6	70	HC-ARF	120	Not stated	10 (integration: 60s)
CH_4_ [49]	1.65	200	HCF	130	Not stated	3.6
C_2_H_2_ [51]	1.53	200	HC-PCF	85	4200 (for filling)	1.4
NH_3_ (this work)	1.51	0.9	MFA	0.4	22 (real-time)	32

HCF, hollow-core fiber; MFA, microfiber array (this work); N/A, not applicated.

## 4 Conclusions

In summary, we introduce two novel acoustic-induced mode-dependent low-frequency shift (AOFS) schemes based on the in-fiber AOI effect. Two AOFSs involve cascading the AIFGs or with the LPFG, to realize the efficient cyclic conversion between transverse core modes (LP_01_ and LP_11_) and the low-frequency shifts of 1.83 MHz and 0.9 MHz, respectively. These schemes also exhibit remarkable characteristics including sideband rejection ratios of over 40 dB, insertion losses of less than 1 dB, and wide dynamic tuning ranges greater than 200 nm. Subsequently, an adiabatically tapered microfiber with 1-μm diameter and 4-mm length was fabricated from standard SMF. Our numerical simulation result shows that 25 % of the pump light is in the form of evanescent field exciting the PT effect. Furthermore, by using the proposed AOFS as the reference arm and the produced microfiber gas cell as the probe arm, a heterodyne interferometric scheme is built to detect the PT effect caused by the gas absorption of the pump-light evanescent field. Via successively demodulating the phase and frequency of the beat signal, an ammonia equivalent detection limit (1*σ*) of 32 ppm with a response time of 22 s can be obtained, as well as 0.24 % instability within 48 pump cycles. By precisely optimizing the two arms’ lengths of the interferometer, the detection limit is expected to reach to sub-ppm level. On this basis, we also investigate other highlights of this PT gas detection system including immunity to ambient noise as well as sensitivity enhancement through pump power boosting and microfiber array application. Compared to other equally sensitive PT gas sensors, our schemes offer a simpler configuration, faster response, and smaller size at a much lower cost. These AOFS schemes we propose are therefore flexible and efficient for practical applications in fiber optic sensing, heterodyne detection, and coherent communications.
